# Epigenome-Wide DNA Methylation in Hearing Ability: New Mechanisms for an Old Problem

**DOI:** 10.1371/journal.pone.0105729

**Published:** 2014-09-03

**Authors:** Lisa E. Wolber, Claire J. Steves, Pei-Chien Tsai, Panos Deloukas, Tim D. Spector, Jordana T. Bell, Frances M. K. Williams

**Affiliations:** 1 Department of Twin Research and Genetic Epidemiology, King's College London, London, United Kingdom; 2 William Harvey Research Institute, Barts and The London School of Medicine and Dentistry, Queen Mary University of London, London, United Kingdom; 3 King Abdulaziz University, Jeddah, Saudi Arabia; 4 Wellcome Trust Sanger Institute, Hinxton, Cambridge, United Kingdom; Universität des Saarlandes, Germany

## Abstract

Epigenetic regulation of gene expression has been shown to change over time and may be associated with environmental exposures in common complex traits. Age-related hearing impairment is a complex disorder, known to be heritable, with heritability estimates of 57–70%. Epigenetic regulation might explain the observed difference in age of onset and magnitude of hearing impairment with age. Epigenetic epidemiology studies using unrelated samples can be limited in their ability to detect small effects, and recent epigenetic findings in twins underscore the power of this well matched study design. We investigated the association between venous blood DNA methylation epigenome-wide and hearing ability. Pure-tone audiometry (PTA) and Illumina HumanMethylation array data were obtained from female twin volunteers enrolled in the TwinsUK register. Two study groups were explored: first, an epigenome-wide association scan (EWAS) was performed in a discovery sample (n = 115 subjects, age range: 47–83 years, Illumina 27 k array), then replication of the top ten associated probes from the discovery EWAS was attempted in a second unrelated sample (n = 203, age range: 41–86 years, Illumina 450 k array). Finally, a set of monozygotic (MZ) twin pairs (n = 21 pairs) within the discovery sample (Illumina 27 k array) was investigated in more detail in an MZ discordance analysis. Hearing ability was strongly associated with DNA methylation levels in the promoter regions of several genes, including *TCF25* (cg01161216, p = 6.6×10^−6^), *FGFR1* (cg15791248, p = 5.7×10^−5^) and *POLE* (cg18877514, p = 6.3×10^−5^). Replication of these results in a second sample confirmed the presence of differential methylation at *TCF25* (p(replication) = 6×10^−5^) and *POLE* (p(replication) = 0.016). In the MZ discordance analysis, twins' intrapair difference in hearing ability correlated with DNA methylation differences at *ACP6* (cg01377755, r = −0.75, p = 1.2×10^−4^) and *MEF2D* (cg08156349, r = −0.75, p = 1.4×10^−4^). Examination of gene expression in skin, suggests an influence of differential methylation on expression, which may account for the variation in hearing ability with age.

## Introduction

The term epigenetics [1] refers to the regulation of gene expression primarily by DNA methylation and changes to DNA folding. Epigenetics plays an important role in gene expression regulation and cell differentiation in the developing organism [2], [3]. While the genetic code is fixed, epigenetic changes may be dynamic and have been shown to change during a lifetime [4]. DNA methylation is one of the most commonly studied epigenetic changes and involves the addition of a methyl-group to the 5th carbon molecule of a cytosine base, generating 5-methyl-cytosine. This stable modification occurs primarily at the CpG dinucleotide, but has also been detected at CpH sides, where H can stand for C, A or T. Each diploid human genome contains on average 10^8^ cytosines, of which about 10^7^ are combined with guanine as CpG dinucleotide [5]. CpG dinucleotides often cluster in CpG-islands in the promoter region of genes. The majority of CpG islands in promoters are unmethylated. DNA methyl-transferases are responsible for *de novo* methylation of DNA [6] and facilitating stable transmission of epigenetic marks during cell division [7].

Epigenetic changes can be influenced by both environmental exposure [4], [8] and genetic variation [9]. Therefore, samples for studying the association of methylation with any given trait should ideally be matched for genetic and environmental variation. This could best be achieved by using family data or monozygotic twins, which are assumed to be genetically identical and well matched for environmental exposures [10]. Family and twin studies have identified differentially methylated regions associated with age [11] and multiple complex traits [4], [12] and have further been used to estimate rates of heritability in DNA methylation [8], [11]. Current advances in technology allow for high-resolution screening of DNA methylation profiles across the genome. Multiple platforms exists, but to date the majority of studies have successfully used the Illumina Infinium HumanMethylation 27 k and HumanMethylation 450 k Bead Chips to assay genome-wide DNA methylation profiles across individuals [11], [13].

Age-related hearing impairment (ARHI) is a common complex trait affecting 46% of the population over the age of 48 [14]. Epigenetic changes in the ageing ear have been proposed to account for age-related changes to hearing ability and syndromic forms of hearing loss [15], [16]. Changes in DNA methylation are influenced by environmental exposure and could therefore provide the essential link between the environment and changes in gene expression. Furthermore, epigenetic changes with age could explain how a previously healthy individual develops hearing loss with age. Several forms of syndromic hearing loss, such as Rett and Stickler syndrome, have been associated with epigenetic change [17], [18], [19]. Here, an epigenome-wide association study (EWAS) of hearing ability was performed, the first EWAS of ARHI to our knowledge. This research aimed to determine significant associations of differentially methylated regions with hearing ability in subjects from the TwinsUK cohort. The most significantly associated CpG sites were replicated in an independent sample and gene expression profiles were investigated at the genes identified.

## Materials and Methods

### Ethics statement

The study was approved by the National Research Ethics service London-Westminster (REC reference number: 07/H0802/84). Fully informed written consent was obtained from all participants prior to study conduction. All research described was conducted according to the rules described in the Declaration of Helsinki.

### Subjects

Hearing data in form of air-conduction PTA was collected from participants of the TwinsUK cohort between 2009 and 2013. Hearing thresholds were determined at frequencies 0.125–8 kHz for each ear according to the recommendations of the British Society of Audiology [20]. Pure-tone audiometry information was summarised by principal component analysis (PCA) [23]. All participants completed a questionnaire covering exposure to environmental risk factors for ARHI and previous ear diseases. Subjects reporting a family history of hereditary hearing loss or signs of conductive hearing loss were excluded from the analysis.

### DNA methylation profiles

Whole blood samples for DNA methylation screening were profiled using two different DNA methylation assays, the Infinium HumanMethylation 27 k BeadChip (26,690 CpG sites) and the Infinium HumanMethylation 450 k BeadChip Kit. In both arrays, the DNA methylation level at a specific CpG site is expressed as the β value, which represents the ratio of the methylated probe signal over the methylated and unmethylated probe signals. The β score ranges from 0 to 1, where 0 indicates absence of methylation and 1 represents a fully methylated CpG site.

The Illumina Infinium HumanMethylation 27 k array measures methylation at 27,578 CpG sites, covering 14,495 genes. This array covers primarily CpG sites located in promoter regions of genes with on average two CpG sites per consensus coding sequence and three to twenty assays per cancer gene [21]. The Illumina Infinium HumanMethylation450 k array covers 485,577 methylation sites in 99% of RefSeq genes (21,231 genes) with an average of 17.2 CpG sites per gene region. [22].

To identify potential confounders of the Illumina Infinium HumanMethylation 27 k array, principal component analysis (PCA) was performed using the normalised DNA methylation values. The first five principal components resulting from this analysis were correlated with following covariates: chronological age, methylation chip and position of sample on the chip. Both methylation chip and position of sample on the chip were significantly correlated with the first two principal components from this analysis and therefore included as fixed effects in further analysis [11]. The same procedure was performed for the Illumina Infinium HumanMethylation 450 k array with covariates age, chip, position of sample on the chip and bisulfite converted DNA concentration levels. Chip, position on the chip and bisulfite converted DNA concentration levels were significantly associated with the first 3 principal components and were therefore included as fixed effects in the linear mixed effects models.

### Epigenome-wide association study

The discovery EWAS of hearing was performed in 115 adult female subjects with available PTA data and Illumina Infinium HumanMethylation 27 k profiles [11]. The DNA methylation profiles used in the EWAS were obtained from 26,690 DNA methylation probes, which mapped uniquely to the human genome (hg18) [9]. After further exclusion of probes mapping to the X-chromosome and probes with missing data, 24,641 autosomal probes remained for the EWAS [11]. The Illumina Infinium HumanMethylation 27 k profiles have been published previously [11]. DNA methylation was transformed to a standard normal distribution per probe using quantile normalisation. To determine the association between hearing ability and DNA methylation a linear mixed effect model was applied. DNA methylation levels at each CpG site were regressed against hearing ability (PC1), with adjustment for age, methylation chip, order of samples on the chip and twin relatedness. To exclude associations with DNA methylation due to covariates other than hearing, the full model was compared to a null model, excluding hearing as a predictor variable. The null and the full model were compared for model fit in an analysis of variance (ANOVA). Only associations where the full model fitted the data significantly better (p<0.05) than the null model were reported. For each significantly associated probe, the effect size (beta), standard error of effect (se) and the p-value from the analysis of variance comparing full and null model were reported. To confirm that the regions of association were not age-dependent differentially methylated regions (age DMRs), models including and excluding age as a fixed effect were compared. Furthermore, associated probes were checked against previously reported age DMRs [11]. In addition, to exclude an underlying association between genetic (rather then epigenetic) variation and PC1, genetic variants in the DMR genomic loci were tested for association with PC1, in a PC1 genome-wide association scan from the TwinsUK cohort (n = 1028). To adjust for multiple testing in the EWAS initially a Bonferroni corrected significance threshold assuming 24,641 independent tests (p = 0.05/24641 = 2.03×10^−6^) was assumed epigenome-wide significant. Furthermore, since the Illumina Infinium HumanMethylation 27 k array contains on average 2 probes per promoter and high levels of co-methylation between nearby probes have previously been reported [9], we also considered 2 additional Bonferroni corrected thresholds: a genome-wide significant threshold correcting for 14,495 independent genes (p = 0.05/14495 = 3.45×10^−6^) and a genome-wide suggestive threshold correcting for 14,495 independent genes (p = 0.1/14495 = 6.90×10^−6^).

### Replication study

The replication sample consisted of 203 females from the TwinsUK registry. For the replication study only the 10 probes most highly associated in the discovery EWAS were investigated, while the remaining 485567 probes from the 450 k array were neglected. The 10 selected probes were examined for replication in the second sample using a linear mixed effect model. DNA methylation was transformed to standard normal per probe using a quantile normalisation. DNA methylation at each CpG site was regressed against hearing ability (PC1) with adjustment for age, methylation chip, order of samples on the chip, bisulfite conversion levels and twin relatedness. To exclude association with DNA methylation due to covariates, the full model was compared to a null model, in which hearing was excluded as a predictor variable. The null and the full models were compared for model fit in an analysis of variance. For each of the 10 probes, the effect size (beta), standard error of effect (se) and the p-value from the analysis of variance comparing full and null model were reported. Replication of association was considered if association was in the same direction and nominally significant (p≤0.05). To confirm that replicating probes were not age-dependent DMRs, models including and excluding age as a fixed effect were compared. To determine the significance and effect of joint association signals in the discovery (27 k) and replication (450 k) samples, a meta-analysis was conducted for the ten most highly associated probes using METAL [23] based on the inverse-variance option.

### DMR validation using methylated DNA immunoprecipitation sequencing (MeDIPseq)

To further validate the findings from the EWAS (27 k) and replication study (450 k) using an alternative technique, the top ranked DMR was also explored using methylated DNA immunoprecipitation followed by high throughput sequencing (MeDIPseq) data. The MeDIPseq validation sample consisted of 46 unrelated healthy females with PTA scores and previously published MeDIPseq profiles [24]. MeDIPseq DNA methylation levels were generated and quantified as previously described [24], and relative methylation scores in a 1 kb region on chr 16 (chr16: 88466501–88467500 on hg 18) overlapping probe cg01161216 (chr16: 88466949 on hg 18) were explored for association with PTA. A linear fixed effect model was applied, where the DNA methylation signal at the locus surrounding the chromosomal position of probe cg01161216 was regressed on hearing ability (PC1), adjusted for age. To exclude an association of DNA methylation with age, the full model was compared to a null model, excluding hearing as a predictor variable. The null and full models were compared for model fit using analysis of variance (ANOVA).

### Whole blood cell subtype heterogeneity

Previous studies have reported that association with DNA methylation measured in whole blood samples can be driven by blood cell subtype heterogeneity [25]. To adjust for this, eosinophil, lymphocyte, neutrophil and monocyte cell counts in the blood samples were included (as fixed effects) in the full and null models for the ten most highly associated probes. 106 out of 115 subjects had complete blood cell counts available and were included in this analysis.

### Exploring methylation changes in monozygotic twins

Monozygotic twin pairs with PTA and Illumina HumanMethylation 27 k data were selected for the MZ discordance analysis (n = 21 pairs). Intra-pair DNA methylation difference per probe was calculated as the difference in DNA methylation residuals (adjusted for chip and position on the chip) between co-twins. DNA methylation residuals were calculated from quantile normalised β values per probe. Differences in DNA methylation were compared to differences in PC1 were using Spearman rank correlation.

### Effect of DNA methylation on gene expression

To investigate the influence of DNA methylation on gene expression, expression levels in skin tissue collected as part of the Multiple Tissue Human Expression Resource (MuTHER) (http://www.muther.ac.uk) were examined [26]. Quantile normalised gene expression in skin was adjusted for experimental batch effect and RNA concentration in the tissue sample and residuals correlated with DNA methylation residuals (adjusted for chip and position on the chip) at the corresponding probes using Pearson correlation. Furthermore, skin expression residuals were correlated with PC1 values, to test for an effect of gene expression on the phenotype.

## Results

### Subjects and phenotypes

Two independent samples with hearing data and DNA methylation profiles were selected from the TwinsUK registry to perform the discovery EWAS (n = 115) using Illumina HumanMethylation 27 k profiles, and the replication EWAS (n = 203) using Illumina HumanMethylation 450 k profiles. Subjects included in the discovery EWAS had a mean age of 56.7 years (±7.9 years of standard deviation from the mean, age range 33–80 years) and included 25 dizygotic twin (DZs) pairs, 21 monozygotic twin (MZs) pairs and 23 singletons. The replication sample included 203 females, comprising 61 MZ twin pairs, 22 DZ twin pairs and 37 unpaired twins (singletons), with a mean age of 63.21 (± 8.87 years of standard deviation from the mean, age range 41–82 years). The discovery and replication samples are summarised in Table 1. Variance in PTA was summarised using principal component analysis, where PC1 represented the threshold shift over all frequencies (0.125–8.0 kHz) and captured 54.25% of the variance. A high PC1 score thus corresponded to reduced hearing ability [27].

**Table 1:** Characteristics of the female TwinsUK samples.

sample	zygosity	n	age at DNA extraction		age at hearing test		PC1 ±sd
			mean ±sd	range	mean ±sd	range	
discovery (27 k)	MZ	42	55.43±6.93	45–68	62.00±6.60	50–72	−0.28±1.47
	DZ	50	57.68±8.88	33–80	64.32±7.68	47–83	0.72±1.53
	singleton	23	56.91±7.35	43–70	64.83±6.12	50–75	0.29±1.78
	Total	115	56.70±7.91	33–80	63.57±7.05	47–83	0.27±1.61
replication (450 k)	MZ	122	55.64±8.83	37–73	63.82±8.79	46–82	0.60±2.13
	DZ	44	52.88±10.59	33–78	60.86±10.58	41–86	0.21±2.48
	singleton	37	55.87±6.20	42–66	63.97±6.28	49–75	0.21±1.74
	Total	203	55.09±8.87	33–78	63.21±8.87	41–86	0.45±2.15
validation (MeDIPseq)	singleton	46	60.02±7.85	41–83	62.28±7.86	43–86	−0.10±2.08

Demographic characteristics of the discovery (27 k DNA methylation bead chip), replication (450 k DNA methylation bead chip) and validation (methylated DNA immunoprecipitation and high throughput sequencing (MeDIPseq) samples are listed. Samples zygosity is shown (monozygotic (MZ) and dizygotic (DZ) twins) as well as unpaired twins (singletons). Demographic measures include number of subjects (n), mean chronological age at DNA extraction in years and age range, as well as mean chronological age at hearing assessment in years. Mean and standard deviation (sd) of hearing principal component 1 (PC1), representing the overall threshold shift in the audiogram, are given.

### DNA methylation profiles

Genome-wide DNA methylation levels were obtained in the set of 115 female twins using the Illumina 27 k array. The majority of autosomal CpG sites included in this analysis were unmethylated (β<0.3, 68.9% of probes), few probes were hemi-methylated (β: 0.3–0.7, 11.2% of probes) or fully methylated (β>0.7, 19.9% of probes).

### Discovery EWAS

**Figure 1:**
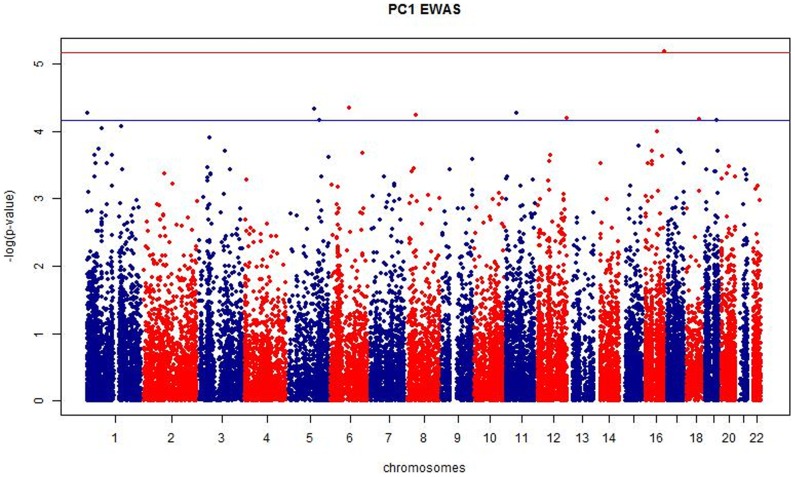
Manhattan Plot of PC1 EWAS results. The manhattan plot depicts the significance of association with PC1 as the negative logarithm of the p-value (-log(p-value)) versus the chromosomal location (chromosomes) for each of the 24,641 tested DNA-methylation probes. The red line defines a Bonferroni adjusted genome-wide suggestive significance threshold of p = 6.9×10^−6^. The ten most highly associated probes are located above the horizontal blue line corresponding to p<6.985×10^−5^.

Genome-wide DNA methylation levels at 24,461 autosomal probes were previously obtained in the set of 115 discovery female twins using the Illumina 27 k array [11] and compared to hearing ability. DNA methylation at 2,519 (out of 24,641) probes was nominally associated (ANOVA p-value≤0.05) with hearing ability for PC1. A Manhattan plot of the EWAS for hearing PC1 is shown in Figure 1 and the strongest signal reached Bonferroni adjusted genome-wide suggestive evidence for association (p = 6.9×10^−6^).

The most highly associated probe was cg01161216 which maps to the promoter region of transcription factor 25 (*TCF25*)(beta±se = −0.245±0.05, p = 6.6×10^−6^). Further associations were observed for CpG sites in the promoter regions of the phosphoglucomutase 3 (*PGM3*) gene (beta±se = −0.26±0.06, p = 4.5×10^−5^), the cysteine dioxygenase type 1 (*CDO1*) gene (beta±se = −0.24±0.06, p = 4.7×10^−5^), the nucleolar complex associated 2 homolog (*NOC2L*) gene (beta±se = −0.20±0.05, p = 5.4×10^−5^), the myosin binding protein C (*MYBPC3*) gene (beta±se = −0.19±0.05, p = 5.4×10^−5^), the fibroblast growth factor receptor 1 (*FGFR1*) gene (beta±se = −0.24±0.06, p = 5.7×10^−5^), the DNA polymerase epsilon catalytic subunit (*POLE*) gene (beta±se = −0.16±0.04, p = 6.3×10^−5^), vacuolar protein sorting 4 homolog B (*VPS4B*) gene (beta±se = 0.20±0.05, p = 6.5×10^−5^), the heterogeneous nuclear ribonucleoprotein A0 (*HNRNPA0*) gene (beta±se = 0.14±0.03, p = 6.9×10^−5^), and probe cg25017250 (beta±se = −0.23±0.06, p = 7.0×10^−5^) mapping to the apolipoprotein C-4 (*APOC4*) gene. The ten most highly associated EWAS probes are listed in Table 2.

**Table 2:** Results of epigenome wide association of hearing PC1 for discovery and replication samples and their meta-analysis.

probe	gene	27 k (n = 115)				450 k (n = 203)				meta-analysis (n = 318)			
		beta	se	p-value	p-value (no age)	beta	se	p-value	p-value (no age)	dir	beta	se	p-value
**cg01161216**	**TCF25**	**−0.24454**	**0.05189**	**6.60E-06**	**4.98E-04**	**−0.12362**	**0.03087**	**8.55E-05**	**1.06E-08**	**− −**	**−0.1552**	**0.0265**	**4.89E-09**
cg25383093	PGM3	−0.26056	0.05631	4.46E-05	7.46E-05	−0.00816	0.03367	8.08E-01	7.66E-02	**−** **−**	−0.0746	0.0289	9.80E-03
cg07644368	CDO1	−0.23819	0.05639	4.67E-05	2.06E-03	0.02340	0.03877	5.50E-01	4.40E-01	**−** +	−0.0606	0.0319	5.80E-02
cg19923810	NOC2L	**−**0.20035	0.04784	5.38E-05	3.24E-05	**−**0.02523	0.03111	4.26E-01	8.16E-01	**−** **−**	**−**0.0773	0.0261	3.05E-03
cg21370143	MYBPC3	**−**0.19031	0.04546	5.44E-05	1.03E-05	**−**0.05170	0.03407	1.30E-01	1.62E-01	**−** **−**	**−**0.1016	0.0273	1.95E-04
cg15791248	FGFR1	**−**0.24243	0.05799	5.73E-05	5.01E-05	**−**0.01488	0.03846	6.96E-01	5.13E-01	**−** **−**	**−**0.0844	0.0321	8.46E-03
**cg18877514**	**POLE**	**−0.16287**	**0.03931**	**6.33E-05**	**9.08E-04**	**−0.06827**	**0.02839**	**1.70E-02**	**2.83E-02**	**− −**	**−0.1007**	**0.0230**	**1.20E-05**
cg05934874	VPS4B	0.19644	0.04751	6.55E-05	8.98E-04	**−**0.08786	0.02993	3.67E-03	1.88E-02	+ **−**	**−**0.0071	0.0253	7.80E-01
cg12241297	HNRNPA0	0.13623	0.03269	6.90E-05	4.52E-04	**−**0.06433	0.02882	2.65E-02	3.18E-02	+ **−**	0.0234	0.0216	2.80E-01
cg25017250	APOC4	**−**0.23167	0.05495	6.98E-05	1.06E-03	**−**0.03949	0.03585	2.71E-01	1.71E-02	**−** **−**	**−**0.0969	0.0300	1.25E-03

The ten most highly associated differentially methylated regions in the discovery EWAS (27 k) are shown. Probes are characterised by the nearest gene, the association effect (beta), standard error of the effect (se) and significance of model fit (p-value). Significance of model fit excluding age as a model parameter (p-value (no age)) is reported for both the discovery (27 k) and replication (450 k) sample. The ten most highly associated probes were taken forward for replication (450 k) with effect (beta) standard error of the effect and significance of model fit (p-value) listed. Results of the meta-analysis are presented as direction of effect (dir, discovery and replication direction shown), combined effect (beta), standard error of the combined effect (se) and significance of the combined association (p-value). Association of DNA methylation and hearing PC1 was replicated for probes cg01161216 and cg18877514 (highlighted in bold).

After exclusion of chronological age as a fixed effect, association of DNA methylation with hearing PC1 remained significant for all of the ten most highly associated probes (Table 2).

### Replication of highly associated EWAS probes

**Figure 2:**
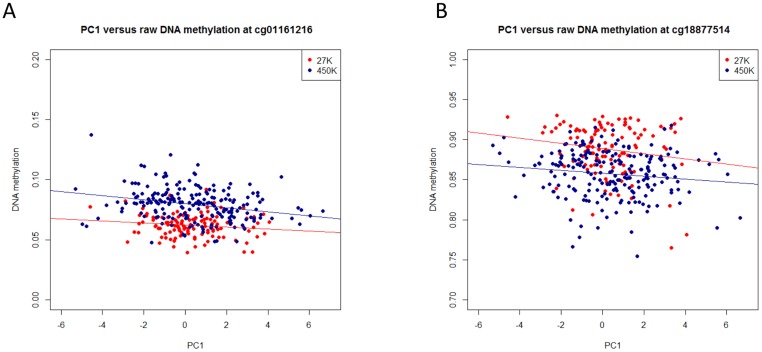
Association of hearing PC1 values and raw DNA methylation at *TCF25* (cg01161216) and *POLE* (cg18877514). A, B Hearing PC1 values were plotted versus raw DNA methylation betas for both the discovery (27 k, red dots) and the replication (450 k, blue dots) samples. Linear regression lines were fitted for both datasets (27 k:red line, 450 k:blue line).

The ten most highly associated CpG probes from the discovery sample were examined in the replication sample (Table 1). Association between DNA methylation and PC1 was replicated at 2 probes - in the promoter regions of genes *TCF25* and *POLE* (Table 2 and depicted in Figure 2). Figure 2 depicts the association between raw methylation betas with hearing PC1 at *TCF25* and *POLE* in the discovery and replication samples. While probe cg01161216 (*TCF25*) was hypomethylated (ß<0.3) in all subjects, probe cg18877514 (*POLE*) was hypermethylated (ß>0.7) (Figure 2). The association between adjusted DNA methylation residuals and PC1 at *TCF25* and *POLE* in the discovery and replication samples can be found in Figure S1. None of the replicating DMRs showed an underlying association of single nuclear polymorphisms with PC1 200 kb up- and downstream of the respective genes (*TCF25*, *POLE*) in a genome-wide association study.

After exclusion of chronological age as a fixed effect, association of DNA methylation with PC1 remained significant at *TCF25* and *POLE* (cg01161216: p(no age) = 1.06×10^−8^; cg18877514 p(no age) = 2.83×10^−2^)(Table 2).

**Figure 3:**
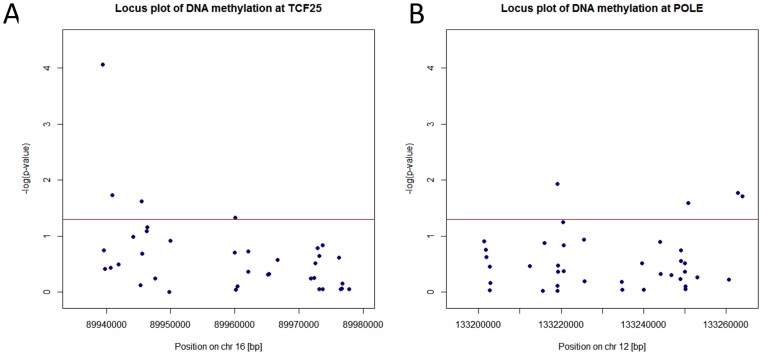
DNA methylation association with hearing PC1 at *TCF25* and *POLE* . Hearing PC1 association with all available Illumina 450 k DNA methylation probes annotated to *TCF25* (A) and *POLE* (B) gene regions according to hg19. Significance of association with hearing PC1 expressed as the negative logarithm of the p-value was plotted against base pair [bp] location for chromosomes 16 and 12, respectively. The red horizontal lines represent the significance threshold for nominal significance (p = 0.05).

To assess the behaviour of additional probes mapping to the *TCF25* and *POLE* loci with respect to hearing, the association between DNA methylation and hearing PC1 was explored for all probes mapping to *TCF25* and *POLE* according to hg19 (Figure 3). According to the locus plots, 3 further nominally significant associated DMRs (p<0.05) mapped to each *TCF25* and *POLE*.

Although DNA methylation was not found significantly associated at 8 out of 10 probes in the replication sample, DNA methylation at five further probes (cg25383093, cg19923810, cg21370143, cg15791248 and cg25017250) showed the same direction of effect as in the discovery sample (Table 2). In the meta-analysis of results from the 27 k and 450 k chips, DNA methylation at 7 out of 10 probes was nominally significantly associated with PC1 (Table 2), with differential DNA methylation at *TCF25* showing the most significant association (cg01161216, p = 4.89×10^−9^)(Table 2).

### Validation of TCF25 using MeDIPseq

To validate the peak EWAS DMR using a different technology, TCF25 DNA methylation levels based on MeDIPseq data were also explored for association with hearing in 46 unrelated females from TwinsUK [24]. The mean age of subjects in the validation sample was 62.28 (±7.86 years of standard deviation from the mean, age range 43–86 years) (Table 1). MeDIPseq DNA methylation levels at a 1 kb locus overlapping probe cg01161216 were selected and compared to PC1. DNA methylation at this locus was significantly associated with hearing PC1 (p = 4.09×10^−2^) and showed the same direction of effect (beta±se = −8.72×10^−6^±4.13×10^−6^) as both the discovery EWAS and replication datasets.

### Blood cell heterogeneity

To account for potential effects of blood cell heterogeneity, the peak DMRs were also explored for association with proportion of eosinophils, lymphocytes, neutrophils and monocytes in a subset of 106 subjects from the discovery sample. The ten most highly associated probes in the discovery EWAS remained significantly associated (p>0.005) with PC1 after adjustment for blood cell heterogeneity.

### Monozygotic co-twin study

MZ discordance analyses were performed in 21 female MZ twin pairs (n = 42) selected from the discovery sample with a mean age of 55.43 years (±6.93 years of standard deviation, age range: 45–68 years) (Table 1). Mean intrapair difference in PC1 was −0.42 (±1.34 sd, range: 3.47 and −2.86). The intra-pair differences in PC1 were compared with intra-pair differences in DNA methylation at 24,641 autosomal CpG sites. Of these, 794 CpG sites were nominally significant (p<0.05). PC1 discordance was most strongly associated with differential methylation at lysophosphatidic acid phosphatase 6 (*ACP6*, cg01377755, r = −0.75, p = 1.2×10^−4^). Further strongly correlated differentially methylated genes included myocyte enhancer factor 2D (MEF2D, cg08156349, r = −0.75, p = 1.4×10^−4^), tachykinin precursor 1 (*TAC1*, cg07550362, r = −0.72, p = 3.3×10^−4^), ATPase family AAA domain-containing 3C (*ATAD3C*, cg27383362, r = −0.70, p = 5.5×10^−4^), brain-specific serine protease 3 (*PRSS12*, cg21208104, r = 0.70, p = 6.3×10^−4^), ADAM metallopeptidase domain 18 (*ADAM18*, cg23566335, r = 0.70, p = 6.5×10^−4^), chromobox homolog 2 (*CBX2*, cg22892904, r = −0.69, p = 7.8×10^−4^), septin 3 (*SEPT3*, cg04283938, r = −0.68, p = 8.6×10^−4^), transmembrane protein 121 (*TMEM121*, cg23886551, r = −0.68, p = 8.6×10^−4^) and torsin family 1 member B (*TOR1B*, cg14299800, r = −0.68, p = 9.1×10^−4^) (Table 3).

**Table 3:** Results of the MZ intra-pair difference association analysis.

MZ pair difference analysis (n = 42)			
probe	gene	rho	p-value
cg01377755	ACP6	−0.753	1.24E-04
cg08156349	MEF2D	−0.749	1.41E-04
cg07550362	TAC1	−0.722	3.25E-04
cg27383362	ATAD3C	−0.703	5.49E-04
cg21208104	PRSS12	0.697	6.26E-04
cg23566335	ADAM18	0.696	6.47E-04
cg22892904	CBX2	−0.688	7.82E-04
cg04283938	SEPT3	−0.684	8.59E-04
cg23886551	TMEM121	−0.684	8.59E-04
cg14299800	TOR1B	−0.682	9.13E-04

This table shows the results for the MZ discordance analysis. Results are listed for the ten most highly correlated probes with corresponding gene, Spearman rank correlation coefficient (rho) and significance of correlation (p-value).

### Influence of DNA methylation on gene expression in skin

**Figure 4:**
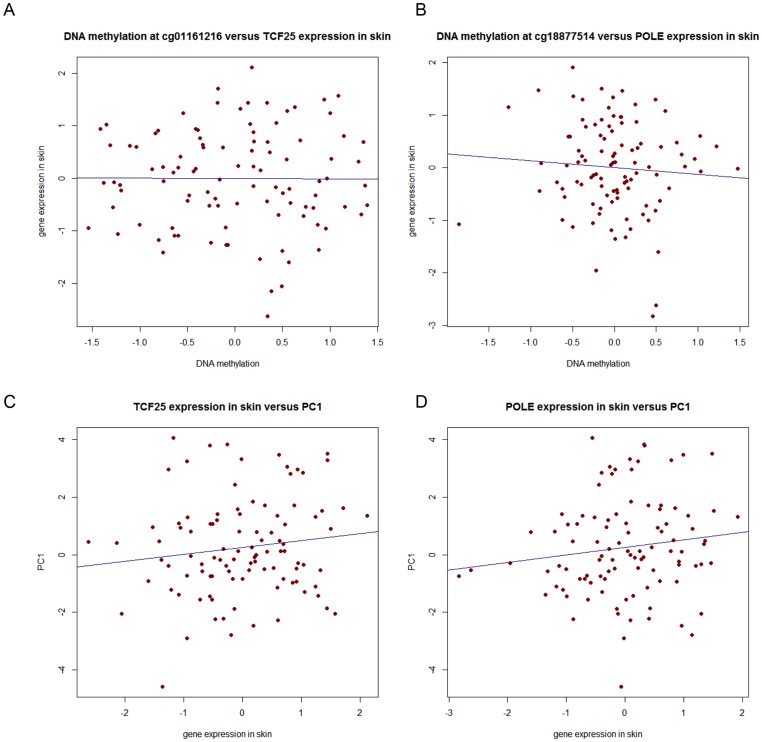
Effect of DNA methylation on gene expression in skin and effect of gene expression on PC1. A . DNA methylation residuals showed a weak negative correlation (r = −0.02) with expression residuals of TCF25 in skin samples. Both quantile normalised DNA methylation betas and quantile normalised gene expression values were adjusted for experimental batch effects (chip and position on the chip for methylation betas and experimental batch and RNA concentration for gene expression profiles) previous to analysis. The regression line (blue line) depicts the linear association between DNA methylation residuals and gene expression residuals. **B**. DNA methylation residuals at probe cg18877514 were weakly negatively correlated (r = −0.06) with *POLE* expression residuals in skin tissue. Both quantile normalised DNA methylation betas and quantile normalised gene expression values were adjusted for experimental batch effects (chip and position on the chip for methylation betas and experimental batch and RNA concentration for gene expression profiles) prior to analysis. The regression line (blue line) depicts the linear association between DNA methylation residuals and gene expression residuals. **C**. *TCF25* expression residuals in skin showed a weak positive correlation (r = 0.12) with PC1. Quantile normalised gene expression values were adjusted for experimental batch effects and RNA concentration. The regression line (blue line) depicts the linear association between gene expression residuals and PC1 values. **D**. *POLE* expression residuals in skin showed a weak positive correlation (r = 0.16) with PC1. Quantile normalised gene expression values were adjusted for experimental batch effects and RNA concentration. The regression line (blue line) depicts the linear association between gene expression residuals and PC1 values.

To investigate the influence of DNA methylation on gene expression, expression profiles in skin were explored because skin originates from the same embryonic tissues as the inner ear, and expression profiles were not available for the cochlea. For 172 individuals with 27 k array data, DNA methylation at the two replicating probes (cg01161216 and cg18877514) was examined for association with gene expression (*TCF25* and *POLE*, respectively). After adjustment of both DNA methylation and skin expression for batch effects, DNA methylation residuals and gene expression residuals showed a weak negative correlation for *TCF25* (r = −0.02) (Figure 4, A) and *POLE* (r = −0.06) (Figure 4, B). In general, DNA methylation in whole blood was only weakly correlated with gene expression in skin tissue. Furthermore, the effect of gene expression levels of *TCF25* and *POLE* on hearing ability was explored. Gene expression showed a weak positive correlation with PC1 values (*TCF25*: r = 0.12; *POLE*: r = 0.16)(Figure 4, Panel C and D).

## Discussion

Changes in DNA methylation have been associated with increasing age and age-related disorders [11]. Here, for the first time the effect of genome-wide DNA methylation on hearing ability was investigated. Genome-wide association and candidate gene studies of hearing ability with age have yet to explain much of the estimated variance in this phenotype. Our approach identified epigenetic changes at a number of genes that were associated with hearing ability, and two of these changes in genes *TCF25* and *POLE* replicated in an independent sample. DNA methylation levels at the strongest signal in *TCF25* validated using an alternative method (MeDIPseq). Hearing PC1 was also strongly associated with DNA methylation at *FGFR1*, a gene known to be essential for maintenance of glial cells and cochlear neurons in the spiral ganglion [28]. These findings suggest that epigenetic changes may account for the variance in severity and age of onset of ARHI.

Nominally significant associations (p<0.05) with PC1, which represents the overall threshold shift in the pure-tone audiogram and hence impaired hearing ability, were identified at 2,519 CpG sites. The ten most highly associated probes remained nominally significant after exclusion of chronological age as a fixed effect in the model, showing that none of these associated probes are age-related differentially methylated probes. Furthermore association remained significant after adjustment for blood cell heterogeneity, indicating that blood cell subtypes were not driving these association signals. Two of the ten signals were replicated in a second independent sample. Changes in DNA methylation in the promoter region of *TCF25* were highly associated with PC1 in both the discovery EWAS and the replication cohort, with meta-analysis p = 4.89×10^−9^. The meta-analysis of the discovery and replication sample was conducted to determine the joint effect of both samples; nevertheless the results of this analysis were driven primarily by the discovery EWAS findings.

As both the discovery and replication data used the same array design from Illumina based on DNA hybridisation an alternative technique, MeDIPseq, was used to validate our findings. MeDIPseq in venous blood from 46 unrelated samples confirmed the association between hearing PC1 and DNA methylation levels at *TCF25* (p = 0.04). This transcription factor belongs to the family of basic helix-loop-helix transcription factors, which is widely expressed in many organs including dorsal root ganglia in mouse embryos [29]; however mouse models of Tcf25 deficiency are not yet available. Over-expression of Tcf25 leads to increased cell death and binding to the X-linked inhibitor of apoptosis protein [30]. Using the ENCODE database [31], probe cg01161216 maps to an area with enhancer and promoter associated histone marks and transcription factor binding sites. Differential expression of *TCF25* might be involved in increased cell death of sensory cells and neurons of the cochlea, resulting in ARHI.

We also identified a differentially methylated DNA methylation probe mapping to the promoter of the *POLE* gene. *POLE* is a DNA polymerase essential for elongation of the leading strand in cell division. In addition, *POLE* is involved in cell cycle regulation and therefore regulates a variety of cellular processes. According to UCSC browser and the ENCODE database [31], probe cg18877514 maps to an area rich in repeating elements with enhancer and promoter associated histone marks and transcription factor binding sites. *Pole* knockout mice with a random gene disruption are embryonic lethal, while *Pole* targeted knock-in mice present with premature death due to cancer and increased tumourigenesis in general [32].

Among the top associations in the discovery EWAS was a DMR in the promoter of *FGFR1*, a gene known to be essential for maintenance of glial cells and cochlear neurons in the spiral ganglion [28]. However, our replication study did not confirm the differential methylation in the promoter of *FGFR1* identified in the discovery EWAS, but the DMR did manifest the same direction of effect. This gene is of particular interest, having been associated with hearing ability in mice [28]. *FGFR1* encodes a fibroblast growth factor receptor, reported to be essential for healthy development of the organ of Corti [33]. Conditional knockout of fibroblast growth factor receptors (*FGFR1* and *FGFR2*) in glial cells in the spiral ganglion resulted in loss of spiral ganglion neurons and age-related hearing loss in mice [28]. Our results show a negative association between DNA methylation at the promoter of *FGFR1* and hearing PC1 (beta = −0.24±0.06 se), indicating that greater methylation (and expected reduced gene expression) of *FGFR1* showed good hearing ability. This direction of effect is not consistent with that observed in mouse cochlea [28].

DNA methylation in the promoter of genes has been associated with repression of gene expression. At our peak DMR in *TCF25*, DNA methylation was found minimally negatively correlated with gene expression in skin (r = −0.02; *POLE*: r = −0.06) – the tissue with the most embryologic similarity to cochlea. However, DNA methylation may be highly tissue specific [34], [35] and in this study DNA methylation was determined from whole blood samples. That gene expression in skin showed a weak positive correlation with hearing PC1 for both *TCF25* (r = 0.12) and *POLE* (r = 0.16), indicates that individuals with decreased hearing ability (high PC1 value) show higher RNA levels of *TCF25* and *POLE* in skin. Whether these findings pertain to RNA expression in the inner ear remains to be determined.

Monozygotic twin pairs are a preferred study sample for epigenetic studies as they are assumed to be genetically identical. In addition, both dizygotic and monozygotic twin pairs show an increased proportion of shared environment due to the nature of their time shared in uterus and upbringing. The MZ discordance analysis was performed to best utilise the unique study sample presented here and for completeness. Nevertheless, the relatively low sample size and restricted discordance within the twin pairs limited the statistical power to detect strong epigenetic effects. Association was examined between intra-pair discordance for PC1 and intra-pair differences in DNA methylation at CpG sites genome-wide. The most highly associated probes were found in the promoters of *ACP6* and *MEF2D*. The function of acid phosphatase 6 is yet unknown and *Acp6* knockout mice are described as phenotypically normal [32]. In contrast, myocyte enhancer factor 2D is a member of the myocyte enhancer factor family of transcription factors, which are involved in neuronal development and differentiation under regulation of class 2 histone deacetylases. *MEF2D* is expressed in mouse cochlear neurons and sensory cells at P15 and was diminished in IGF knockout mice, which show sensorineural hearing loss [36]. The data indicate that *MEF2D* is a plausible candidate gene for ARHI and may be under epigenetic control.

Our study has several strengths and limitations. DNA methylation is likely to play an important role in gene expression contributing to important phenotypic differences between tissues, between individuals and with age. Methods of analysis of methylation data are in their infancy: there are many important covariates to be considered. We elected to remove one of these, gender, by confining our studies to females, which predominate in the TwinsUK database. Thus our results pertain to women and may not extrapolate to men. Strengths included ability to exclude age and blood cell heterogeneity as potential confounders. The high proportion of related individuals in this sample reduced both the genetic and environmental variance compared to a population sample of unrelated individuals. Although the discovery and replication datasets were well matched for gender, ethnicity, age and hearing ability, the replication sample included by chance a higher proportion of monozygotic twin pairs (450 k sample: 60% MZs) compared to the discovery sample (27 k sample: 37% MZs), which might have resulted in the reduced significance of associations obtained in the replication sample. Association in the EWAS did not reach epigenome-wide significance by Bonferroni corrected significance levels (considering 24,641 independent tests: p≤2.03×10^−6^). However, DNA methylation of neighbouring CpG sites is unlikely to be independent thus a Bonferroni correction may be considered overly stringent. Taking co-methylation into account by correcting for the number of genes, the peak DMR in *TCF25* surpassed genome-wide suggestive evidence for association. Further limitations of the study included the choice of tissue: although DNA methylation is tissue specific, whole blood samples were used as an initial approach to this investigation because they were readily available and inner ear tissue from humans was not. In addition, discordance in hearing ability within TwinsUK monozygotic twin pairs was relatively limited. Finally, it should be noted that this study makes no assumptions about causal relationships between DNA methylation and ARHI. A longitudinal study design would be required to confirm that the methylation changes inferred by these results predated the onset of hearing impairment.

In conclusion, this is the first study investigating the association between hearing ability with age and DNA methylation genome-wide in humans. Strong associations with DNA methylation in the promoters of 10 genes were identified, of which two (*TCF25* and *POLE*) were replicated in an independent cohort. Functional studies will be required to explore further the effect of epigenetic regulation of these genes in ARHI. Proof of epigenetic regulation in the development of ARHI would highlight the impact of changes in DNA methylation with age and therefore be of fundamental importance not only for hearing loss but also other age-related disorders.

## Supporting Information

Data S1 Replication study dataset (450 k). The replication study dataset shows DNA methylation betas at the 10 probes selected for replication from the Illumina HumanMethylation 450 k Beadchip for all subjects of the replication study (n = 203). Each subject has been allocated an anonymous identification number (PUBLIC.ID) and is presented by a family-identification number to identify twin siblings (family_zygosity), the age at hearing test (Age_pta), their hearing PC1 value (PC1_unadjusted), gender (SEX), age at DNA extraction (DNA_age), bisulfite conversion values (BSCng_ul), DNA methylation chip and order on the chip (chip and chipo, respectively).
(TXT)

Figure S1 Association of hearing PC1 values and DNA methylation residuals at *TCF25* (cg01161216) and *POLE* (cg18877514). **A, B** Hearing PC1 values were plotted versus DNA methylation beta residuals (adjusted for age, batch effects and relatedness) for both the discovery (27 k, red dots) and the replication (450 k, blue dots) samples. A linear regression lines was fitted for both datasets (27 k:red line, 450 k:blue line).
(TIF)
