# Supplementary figures and images for: Epigenome-Wide DNA Methylation in Hearing Ability: New Mechanisms for an Old Problem
Source: PLoS One. 2014 Sep 3;9(9):e105729. doi: 10.1371/journal.pone.0105729 (PMC4153547; doi:10.1371/journal.pone.0105729)

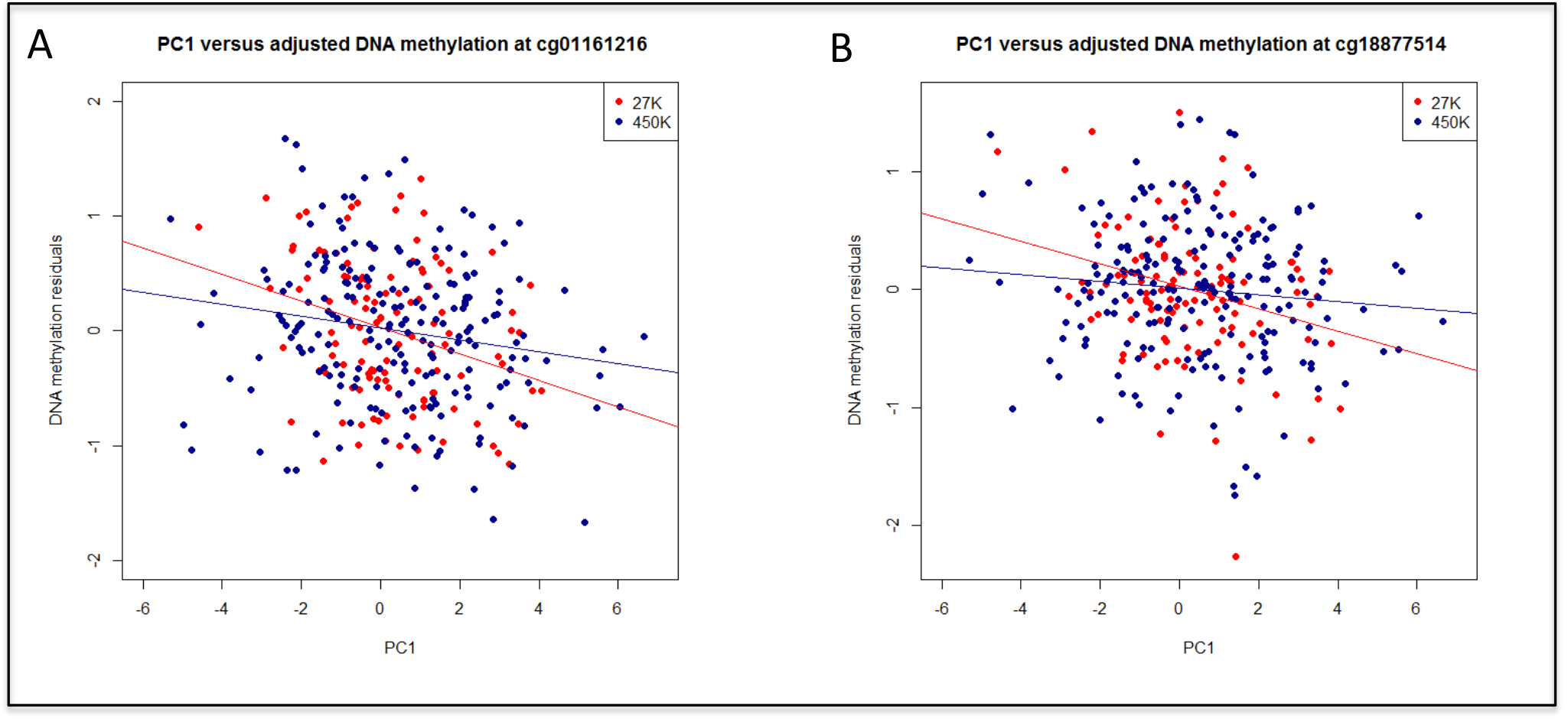

Supplement: Figure S1 — Association of hearing PC1 values and DNA methylation residuals at TCF25 (cg01161216) and POLE (cg18877514). A, B Hearing PC1 values were plotted versus DNA methylation beta residuals (adjusted for age, batch effects and relatedness) for both the discovery (27 k, red dots) and the replication (450 k, blue dots) samples. A linear regression lines was fitted for both datasets (27 k:red line, 450 k:blue line). (TIF) [file pone.0105729.s002.tif]
